# Feasibility Study on the Fused Filaments of Injection-Molding-Grade Poly(Ethylene Terephthalate) for 3D Printing

**DOI:** 10.3390/polym14112276

**Published:** 2022-06-02

**Authors:** Hsi-Hsun Tsai, Shao-Jung Wu, Yu-De Wu, Wei-Zheng Hong

**Affiliations:** 1 Department of Mechanical Engineering, Ming Chi University of Technology, New Taipei City 24301, Taiwan; hhtsai@mail.mcut.edu.tw (H.-H.T.); m09118006@mail2.mcut.edu.tw (Y.-D.W.); 2 Department of Chemical Engineering, Ming Chi University of Technology, New Taipei City 24301, Taiwan; m10138115@mail2.mcut.edu.tw

**Keywords:** fused filaments, 3D printing, injection molding, crystallinity, tensile strength

## Abstract

Unlike that of glycol-modified Poly(ethylene terephthalate) (PETG), the crystallinity of PET can be post-adjusted to enhance the mechanical properties of 3D-printed parts such as food-contact tableware and bio-implants. The aforementioned PET material could be 3D printed to produce the desired parts for performance evaluation before mass production by injection molding. In this study, using differential scanning calorimetry (DSC), we examined the pellets, extruded filament, and printed specimen to identify variations in melting and crystalline temperatures, as well as crystallinity. It was also shown by Thermogravimetric Analyzer (TGA) that the addition of talcum powder increased the thermal stability of filament and resulted in an interaction between the fillers and polymer matrix. The crystallinities of the filament and printed specimen were then compared with the yield strengths and Young’s moduli to confirm the effects of the decreased molecular weight of the extruded PET filament. The talcum powder effectively improved the viscosity of the PET melted during the extrusion process for the filament and then enhanced the crystallinity of the PET, thereby achieving a significantly higher Young’s modulus. The printed PET specimen presented an excellent yield strength of 25 MPa and ductile properties with strain-at-break values of 30%, successfully indicating potential applications in food-contact tableware and bio-implants.

## 1. Introduction

Poly(ethylene terephthalate) (PET) has excellent mechanical, chemical, and thermal properties for use in bottles, packaging films, and textile fibers. PET can exist in both amorphous and semi-crystalline forms, depending on the material’s processing and the thermal conditions. PET has unique properties such as heat resistance, chemical resistance, toughness, and rigidity, which together make PET a feasible filament for application in three-dimensional (3D) printing. However, the low viscosity and low melt strength of PET create challenges in using PET as a 3D filament. The melt strength depends on the molecular weight of the polymer, which provides a stable diameter and cross-section geometry, as well as traction force, during the extrusion of the filament. The 3D filament must also have a certain fluidity so that it can flow at a certain rate in a molten state in the heating head of the 3D printer, which makes the preparation of 3D filaments from PET challenging.

By enhancing the crystallization rate, PET shows opaque, regularly arranged molecules and higher density. Therefore, crystalline PET has high strength and heat resistance, while amorphous PET is transparent and has higher impact strength when bending [1,2,3]. Unlike glycol-modified PET (PETG), which does not crystallize and is treated as an amorphous resin [4], PET can provide higher heat resistance, strength, and toughness under certain rates of crystallization for biomedical implants [5] and food-contact tableware [6]. To successfully derive prototypes during the evaluation of the design phase, an organic polymer filament could be efficiently manufactured by 3D printing. The most common commercially available filaments are PETG [7], acrylonitrile butadiene styrene (ABS), and polylactic acid (PLA) [8]. However, these materials do not satisfy the true evaluation of mechanical performance in the design phase, such as the aforementioned biomedical implants and food contact tableware. Semi-crystalline polymers can fulfill the needs of mechanical performance through the crystallization behavior exerting profound influences on the molecular diffusion and entanglements, as well as the crystalline structures at the interface of the bond layers. The onset of crystallization induces a dramatic increase in melt viscosity and hinders polymer inter-diffusion [9].

Plastic filaments using amorphous polymers are widely applied to facilitate additive manufacturing. Mechanical strength is acquired from semi-crystalline polymers, polyether ether ketone (PEEK) [10,11], an advanced engineering plastic, and PET [12,13,14]; these materials are, therefore, essential in medical, chemical, aerospace, and electronics applications using fused filament fabrication. PEEK is a thermoplastic biomaterial with good thermal resistance, stability, and mechanical properties. This material has an extrusion temperature that varies from 340 to 440 °C, tensile strength equal to 100 MPa, and flexural strength equal to 170 MPa [11]. However, the high cost of PEEK filament for 3D printing makes this material suitable only for valuable applications in bio-implants. Previous studies explored the recycling of polymeric materials for the production of filaments in 3D printing, such as PET, ABS, PLA, polypropylene (PP), nylon (PA), polystyrene (PS), polyethylene (PE), acrylonitrile styrene acrylate (ASA), polyvinylchloride (PVC), high-impact polystyrene (HiPS), polycarbonate, high-density and low-density polyethylene (HDPE, LDPE), and thermoplastic polyurethanes (TPU) [12,15,16,17]. The recycling phases for filament fabrication involve cleaning, grinding, melting, extrusion, and measuring. Moreover, the addition of binders, organic solvents, and re-enforcing additives may have a niche commercial market. However, an original polymer that has reliable physical and chemical properties is needed. Camden et al. [18] investigated blends of PET/PP/polypropylene-graft-maleic anhydride (PET/PP/PP-g-MA) at ratios of 70/20/10 and 75/20/5 for fused filament fabrication. The authors found that the compatibilized blends reduced geometric deviations from the targeted design dimensions but without improvements to tensile strength. The authors also revealed that multiple layers should be deposited before the crystallization halftime to ensure deposition without warping, which can cause print failure. In this study, blending PET and PP was found to achieve the viscosity needed for 3D printing; nevertheless, the strength never changed. A post-processing approach could be utilized to enhance the percent crystallinity of 3D-printed parts, which may increase part strength.

The viscosity of melting PET for injection molding is much lower than that for the plastic extrusion process. Hence, using injection-molding-grade PET for the extrusion of PET filament produces higher geometric deviations in the diameter between the extrusion mold and the filament. This higher melt viscosity can produce challenges during the extrusion process. As an inorganic additive, calcium carbonate (CaCO_3_) is commonly used as a filler to moderate the processing viscosity of melting polymeric materials [19]. Additionally, the viscosity and flow behaviors of the molten polymer during extrusion can inform printability and dimensional accuracy in 3D printing [20]. The talcum would balance the viscosity of molten polymer to meet the dimensional accuracy of filament in the extrusion process and then the accuracy of the printed part in 3D printing.

The use of talcum, which acts as a nucleating agent, yields better results in terms of thermal stability and tensile strength [21,22]. Adding an inorganic additive could increase the melting viscosity of the PET filament and increase the crystallinity percentage of printed parts, but this PET containing an additive would differ from injection-molding-grade PET material. Hence, the subject of the present study is the use of injection-molded PET pellets to fabricate PET filaments for 3D printing, which can provide an assessment of material properties prior to injection molding for mass production. Accurate temperature control is fundamental for the extrusion of this injection-molding-grade PET to filament. Not only small geometric deviations of the filament in additively manufactured parts but also proper viscosity of the melting filament is necessary to ensure the PET filament’s successful implementation on injection-molding-grade PET printed parts for the evaluation of mechanical performance. We also analyze the crystallinity percentages of the filament and 3D-printed parts with respect to the tensile strength in the experiments.

## 2. Materials and Methods

### 2.1. Materials and Filament Fabrication

In this study, Tairilin PET 4112XX was supplied by Nan Ya Plastics, Taiwan, for the fabrication of the filament with injection-molding-grade PET pellets. Talcum powder (commercially labeled as SP-3000) was purchased from Jatery Chemical Co., LTD. (New Taipei City, Taiwan). The average diameter of the talcum particle is 4.5 μm under the mesh number of 3000. The PET pellets were dried for 4 h at 120 °C in a dryer and then extruded using a twin-screw extruder with four heating zones along with the profile of the screw. To increase the solidification rate of the printed specimen, 6 % of talcum powder was added to the PET pellets before the extrusion process [23,24]. The PET pellets were allowed to equilibrate for at least 15 min before beginning extrusion. Filament with the 1.75 mm target diameter was produced by manually matching the screw speed of the extruder, PHM-30, Pin Ying Machine, Kaohsiung, Taiwan [25]. The diameter of the extruder is 1.5 mm for fabrication of the filament, with a diameter of 1.75 mm. The extruded filament from the extruder nozzle to the filament spooler is continuously cooled under ambient temperature by a natural convection effect and then cooled by a water bath. The temperature profile of the four heating zones within the barrel was set to 175 (hopper zone)/195/225/245 °C (die zone) with a screw rotation speed of 50 rpm. The obtained PET filament was then quenched in two room temperature water baths, dried, and coiled into a roll.

### 2.2. Melt Flow Index

Under the influence of a defined temperature and force, the melt flow index (MFI) is important to determine the properties of the polymer necessary to flow through a standard die. The equipment used was a GOTECH Melt Flow Indexer, model GT-7100-MIBH. In this study, a standard load of 2.16 kg was applied on PET grades at a die temperature of 250 to 270 °C, and the flow of material was derived for 10 min.

### 2.3. Viscosimetry

Intrinsic viscosity (IV) was used to represent the molecular weight of PET pellets, filaments, and 3D printed specimens in this study. The intrinsic viscosity (IV) was measured in a 1:1 mixture (by weight) of phenol and 1, 1, 2, 2-tetrachloroethane at 25 °C using a 1C Ubbelhode capillary viscometer. The samples were accurately weighed, then dissolved at a concentration up to 1 g dL^−1^ and filtered.

### 2.4. Thermogravimetry Analysis (TGA)

TGA was performed with an SDT Q600 TA thermogravimetric analyzer under air atmosphere flow. The sample was heated from 40 °C to 800 °C at a rate of 10 °C/min. All materials were dried at 100 °C for 4 h using a vacuum oven before TGA measurement. The percentage of the added talcum powder within the PET filament would be derived by TGA.

### 2.5. Differential Scanning Calorimetry (DSC) Analysis

DSC measurements were performed using a TA Instruments Discovery DSC 25 under a nitrogen atmosphere flow. The temperature scale and the energy output of the calorimeter were calibrated using indium (*T_m, onset_* = 156.6 °C and Δ*H_m_* = 28.6 J/g). Samples of PET pellets, extruded 3D printing filament, and printed specimen are prepared about 5 mg in weight and enclosed in aluminum DSC capsules, respectively. The polymers were dried at 100 °C for 4 h using a vacuum oven to remove moisture before any measurements. Nonisothermal DSC measurements were performed using the following sequence: (1) Heating was first performed from 30 to 300 °C at a rate of 10 °C/min. (2) The previous thermal history was then erased by keeping the samples at 300 °C for 1 min. (3) Next, the molten sample was cooled to 30 °C at a controlled rate of 10 °C/min. (4) The sample was then maintained at 30 °C for 1 min to equilibrate the temperature, followed by (5) heating from 30 to 300 °C at 10 °C/min. From these measurements, all relevant transition temperatures and enthalpies were obtained.

### 2.6. Printed Specimens

**Figure 1:**
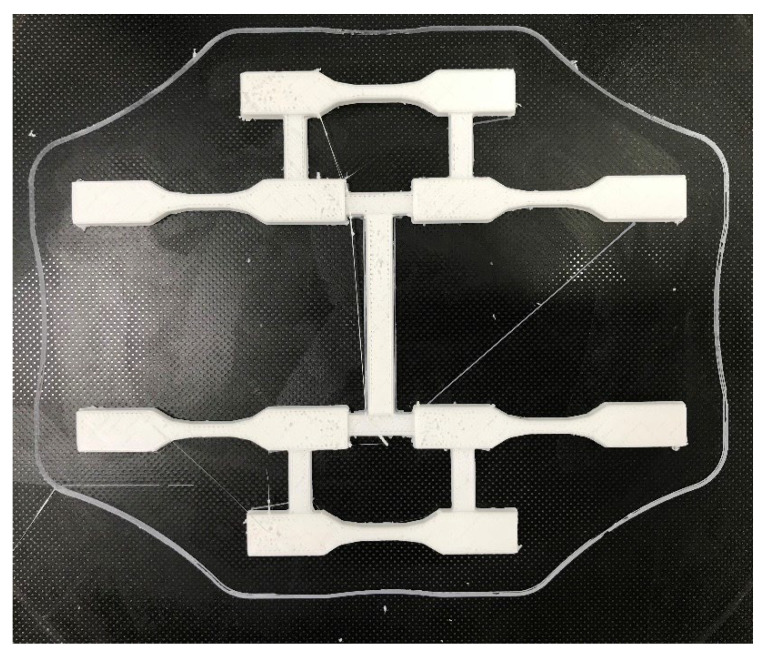
ASTM D638 specimens printed using a 3D printer.

ASTM D638 type IV with a 2 mm thickness was printed using a 3D printer of CR-10 smart, Creality, as shown in Figure 1. The setting parameters of printing were as follows. The thickness of the printed layer was 0.2 mm, the nozzle temperature of the printer was 255 ℃, the table temperature of printer was 50 ℃, and the fill rate was 100%.

### 2.7. Strength Testing

**Figure 2:**
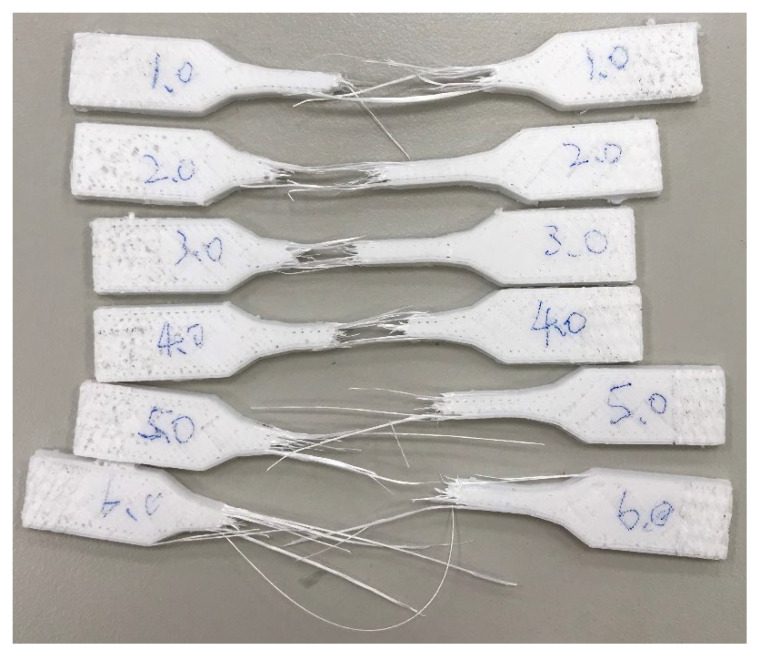
Breakage of the tensile specimens.

Tensile tests were performed in a universal testing machine (Instron 5569, Norwood, MA, USA). The Young’s modulus (*E*), yield strength (σ_y_), and strain at break (ε_b_) were obtained from the load–displacement curves using crosshead speeds of 5 mm/min. A minimum of five tensile specimens by way of 3D printing were tested for each reported value. Figure 2 shows a set of fractured samples after the tensile test.

## 3. Results and Discussion

### 3.1. Viscosity

Extrusion of the 3D filament was carried out at a specific extrusion temperature with the extrusion temperature under an appropriate screw speed. The diameter and tolerance of the extruded filament were 1.75 ± 0.15 mm. The extrusion hole size of the extrusion die was not consistent with the actual extrusion wire size because the hole size is related to the material temperature, mold temperature, and extrusion speed. Therefore, in the extrusion experiment, the diameter of the extrusion wire must be adjusted to within the necessary tolerance based on trial and error. However, the diameter of the wire rod is affected by the screw speed. When the screw speed increases, the wire rod will experience expansion problems. Once a proportion of inorganic talcum powder is added, the viscosity of the melting PET may increase. Generally, the added fillers could act as physical bonding nodes, increase the chain entanglements and restrict the movement of polymer chains, thus enhancing the viscoelasticity of the melt [26,27]. In addition, the addition of talcum powder also increases the crystallization rate of PET and the crystallinity of PET. The crystalline region could be regarded as a physical cross-linking point to improve the melt strength, which is beneficial to the manufacture of filaments. It is then necessary to adjust the extrusion temperature, screw speed, and wire diameter to the required specifications.

**Figure 3:**
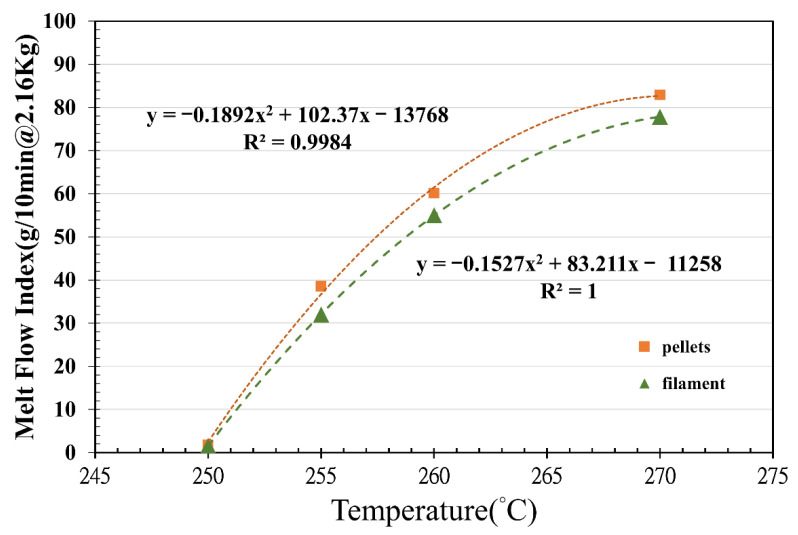
Melt flow index of Tairilin PET 4112XX pellets and filament with respect to heating temperature.

A melt flow indexer, GT-7100-MIBH of GOTECH, was used in this study to evaluate the flow of the melt polymer, and the PET pellets were analyzed following the ASTM Standard Test D1238 from 250 to 270 °C under a 2.16 kg load (ASTM D1238). As shown in Figure 3, the melt flow index (M.I.) of the virgin PET pellet of Tairilin PET 4112XX presented an exponential relationship with the melt temperature. This PET pellet was of injection molding grade and subjected to a higher M.I. under the recommended melt temperature of 255 °C. The M.I. of the virgin pellet has a parabolic relationship with respect to the temperature. After adding the talcum powder into the PET pellet for extrusion of 3D filament, the M. I. of the 3D printing filament is lower than the one of the virgin pellet under the same temperature. Tairilin 4112XX PET pellet has excellent viscosity (above 255 °C) for injection molding. However, the general M.I. level of the required extrusion condition for 3D printing filament is lower than 4.0. Accurate control should also be ensured for the extrusion of this PET material. In Figure 3, although the fabrication process of the filament causes PET chain scissions [28] and gives low viscosity, a lower M.I. of the filament is observed than that of the pellets due to the fact that talcum powder may enhance the melt viscosity of the fused filament.

### 3.2. Nonisothermal Crystallization via DSC

**Figure 4:**
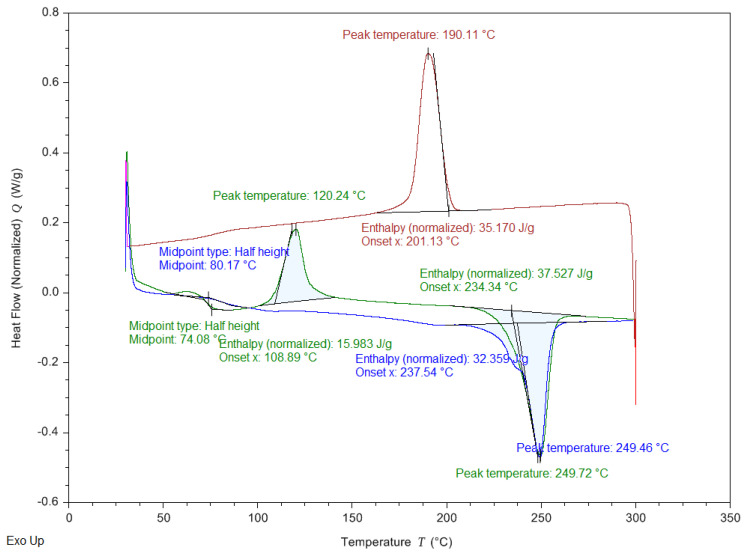
DSC scans of the printed specimen: first heating run (green curve), cooling run from the melt (red curve), and subsequent heating run (orange curve). The cooling and heating rates were 10 °C/min in all cases.

The crystallinity and melting behavior were studied using calorimetric scans under a nitrogen atmosphere. A heating/cooling/heating program was used in a temperature range between 30 and 300 °C. Figure 4 displays the two melting endotherms of printed specimens observed via DSC at 249.72 and 249.46 °C (second heating). The analogous crystallization exotherm was observed to have a peak temperature of 190.11 °C. The melting temperatures (*T*_m_), melting enthalpies (Δ*H*_m_), and glass transition temperatures (*T*_g_) were directly calculated from the thermograms; the degree of crystallinity (*X*_C_) was similarly calculated as *X*_C_ = Δ*H*_m_/[(1 − m_filler_)Δ*H*_m0_], where Δ*H*_m_ is the melting enthalpy, m_filler_ is the ratio of theoretical content in weight and Δ*H*_m0_ is the theoretical melting enthalpy of the fully crystalline PET. In this study, the theoretical melting enthalpy of fully crystalline Tairilin 4112XX PET was assumed to be 140 J/g [29]. In all cases, the degree of crystallinity (*X*_C_) was higher for the reprocessed samples than for PET 4112XX and also progressively increased with each mechanical and thermal process. The chain scission caused by thermo-mechanical degradation at the extruder may produce an increase in crystallinity and, as a result, a loss in mechanical and chemical properties.

**Figure 5:**
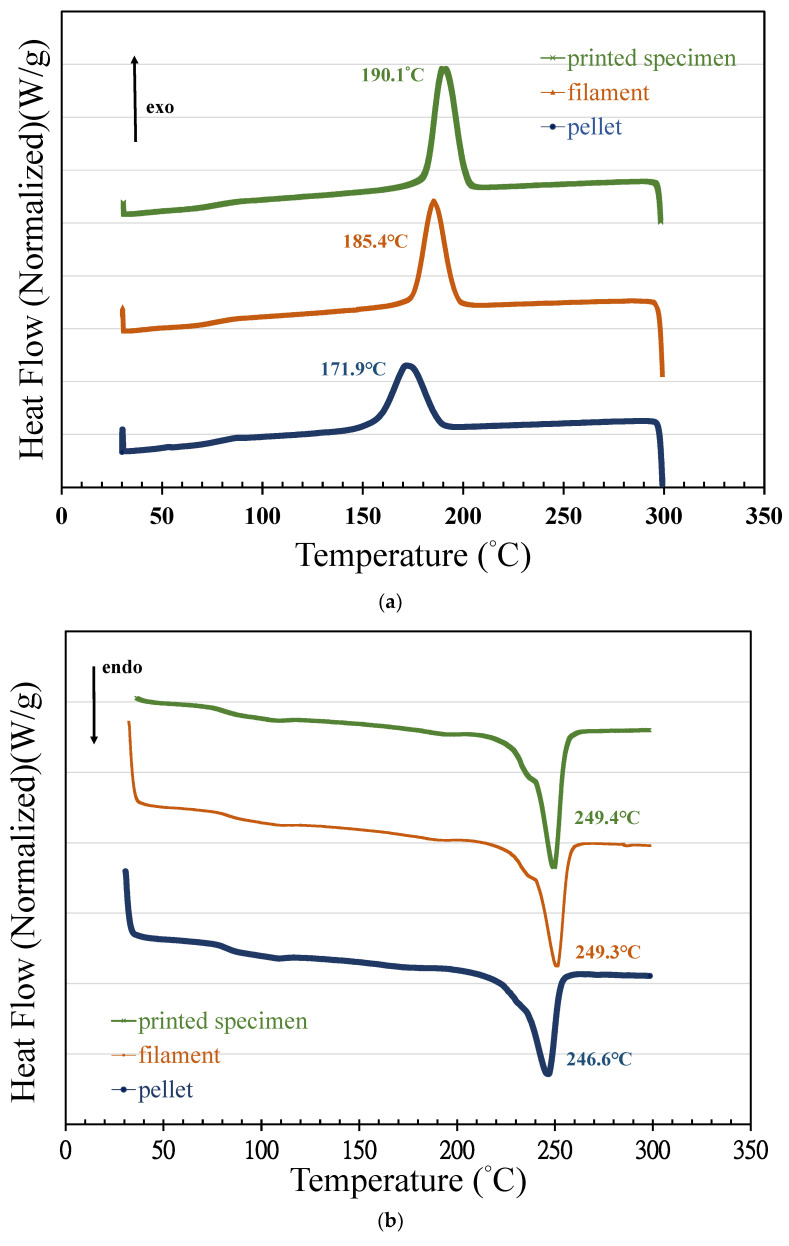
DSC scans: (**a**) cooling runs from the melted pellets, filament, and printed specimen; (**b**) second heating runs based on DSC scans of the melted pellets, filament, and printed specimen.

To compare the temperatures of melting and crystallization of the PET pellets, extruded 3D printing filament, and printed specimen, DSC analysis was used to scan the heat flow variations. Parameters such as melting and crystallization temperature and enthalpy, as well as crystallinity, were the main focuses of the study. The influence of reprocessing on crystallization behavior was investigated using the cooling thermograms shown in Figure 5a. The exotherms of the pellet, extruded filament, and printed specimen presented peak temperatures of 171.9, 185.4, and 190.11 °C, respectively. The previous thermal histories of materials were erased by the first heating scan, after which the chains were free to rearrange into new crystalline stages. The cooling thermograms also appeared sharper and shifted to higher temperatures, suggesting that the crystallization process occurred at a higher temperature for the filaments. This phenomenon can be attributed to the fact that the addition of talcum powder as a nucleating agent increased the crystallization rate of PET during the cooling process. The recorded crystallization temperature (*T*_C_) and crystallization enthalpy (Δ*H*_C_) are outlined in Table 1.

Figure 5b shows the curves of the second heating scan used to study the influence of PET processing history on melting behavior. Based on the melting endotherms, the melting temperatures of the virgin PET pellets, extruded filament, and printed specimen gradually decreased with respect to the history of the mechanical and thermal process. The melting enthalpy (Δ*H*_m_) and glass transition temperature (*T*_g_) were calculated directly from the thermogram, and the degree of crystallinity (*X*_c_) was obtained from the melting enthalpy Δ*H*_m_ and the theoretical melting enthalpy of fully crystalline PET. As shown in Table 1, the crystallinity (*X*_C_) of the filament and the printed specimen was higher than that of the PET pellets. There are two possible reasons for this increase in PET crystallinity: One is that the addition of talcum powder increased crystallinity; the other is that chain scission caused by thermomechanical degradation after mechanical and thermal processing histories led to an increase in crystallinity. As the number of free chains induced by thermomechanical degradation is increasing, the tendency of the smaller chains to fit among the larger ones promotes the packing of these chains in crystalline domains. An increase in the degree of crystallinity is observed during consecutive extrusion cycles [30]. The cooling thermograms of crystallization also show that crystal growth could have been accelerated by reducing the molecular weight of the PET.

In order to understand the change in molecular weight of each sample, the viscosity average molecular weight ($M_{V}^{\;}$) can be calculated using the Mark–Houwink equation,$\;\eta =KM_{V}^{\;\alpha }$, where *K* and *α* are the Mark–Houwink constants and their values are 2.1 × 10^−4^ dL g^−1^ and 0.82, respectively [31]. As shown in Table 2, the intrinsic viscosity and viscosity average molecular weight of the PET pellets is the highest, followed by the filaments, and the lowest is the 3D printed specimens. The decrease in the molecular weight of PET was mainly related to the thermal scissoring effect of the extrusion process. In addition, PET molecular chain scission leads to local molecular weight reduction, which could promote the PET crystallization rate [32].

### 3.3. Thermogravimetric Analysis

**Figure 6:**
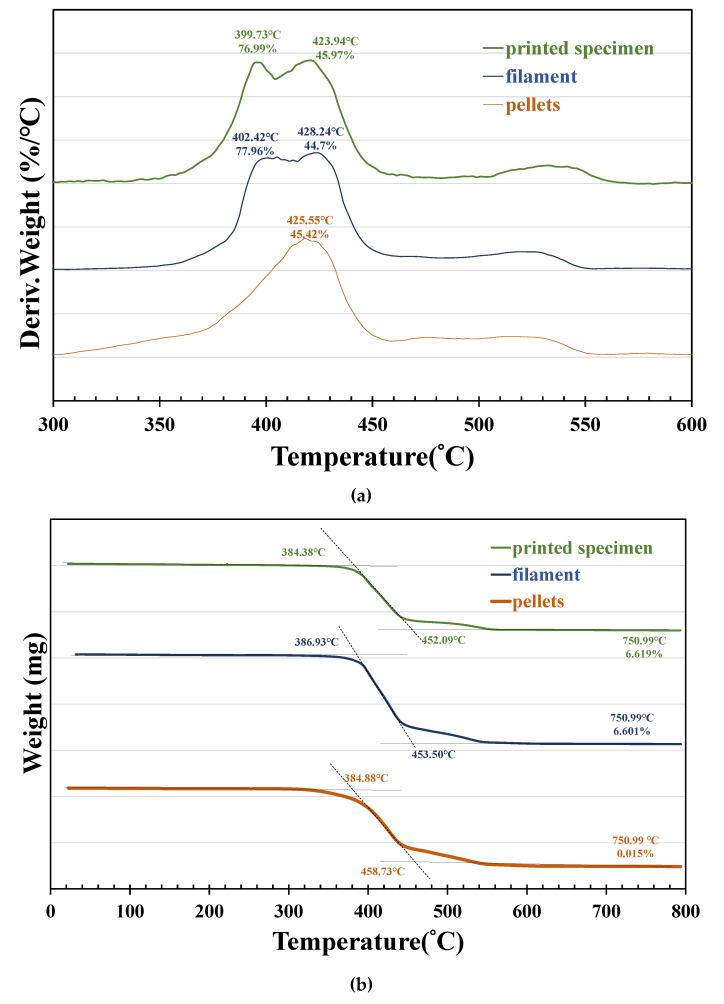
TGA profiles of the pellets, filament, and printed specimen: (**a**) DTG curve; (**b**) TGA curve.

The TGA weight loss data and DTG plots for the PET samples obtained under an air atmosphere are shown in Figure 6a,b. Complete conversion of the PET pellets was observed under oxygenated TGA due to the combustion of char at high temperatures. The PET filaments and 3D-printed materials presented a weight retention percentage that was 6.6% higher than that of the PET pellets in the high-temperature region, indicating that the residual component was talcum powder. The values of the onset temperature (T_o_), peak degradation temperature (T_p_), and residual weight left after each TGA experiment are given in Table 3. The onset temperature of the filament was found to be slightly higher than that of the pellets, with its peak degradation temperature presenting a double peak. It is speculated that the thermal stability of the filament was increased by talcum powder and indicates that there is an interaction between the particles and polymer matrix [30]. Although the crystallinity of the 3D printed samples increased slightly, the thermal degradation temperature had exceeded its melting point and was in a molten state, and its onset temperature and peak degradation temperature were slightly lower than those of the filament, mainly due to molecular chain session during reprocessing.

### 3.4. Mechanical Properties

**Figure 7:**
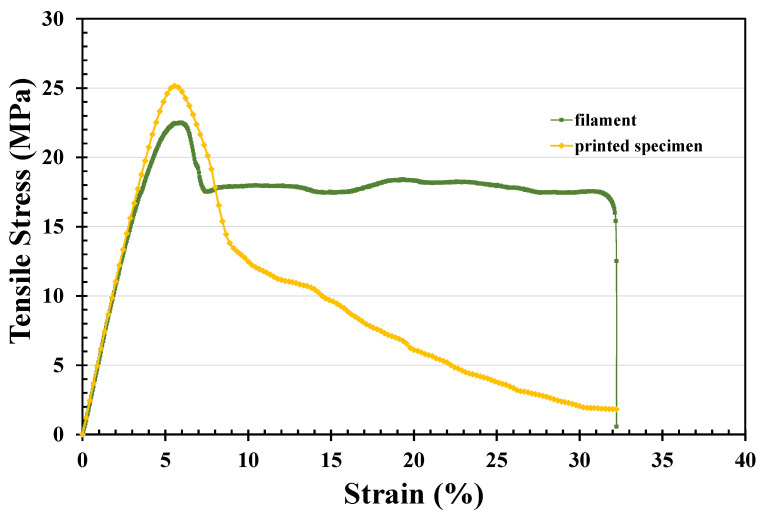
Stress–strain curves of the extruded filament (diameter, 1.72 mm; measured length, 37.5 mm; tension rate, 5 mm/min) and the printed specimen (ASTM D638; section, 3.16 × 2.7 mm; measured length, 15 mm; tension rate, 5 mm/min).

**Figure 8:**
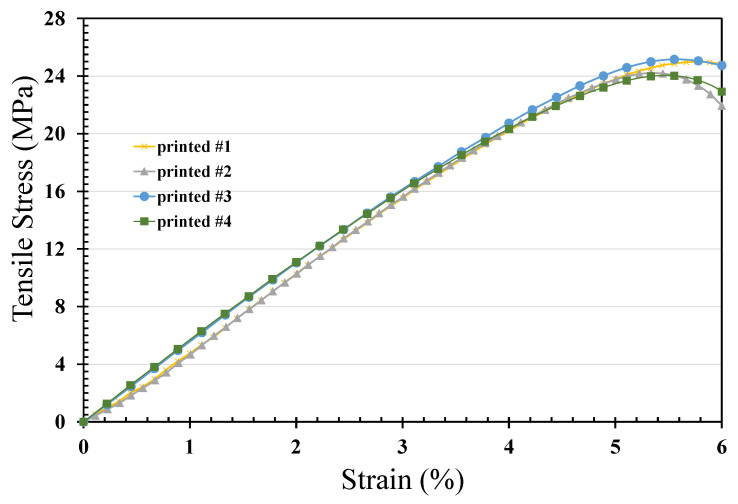
Yield strength of the printed specimen (ASTM D638; section, 3.16ⅹ2.7 mm; measured length, 15 mm; tension rate, 5 mm/min).

In this study, the filament was extruded from the melt pellets. This process can cut the chain scission of PET and yield lower strength. However, higher crystalline rates were observed in the printed specimen and filament, which produced higher strength in the processed PET. Figure 7 shows typical stress–strain curves of extruded PET filament, indicating a yield strength of 23 MPa. The tension testing of the printed PET specimen is also depicted and shows the yield strength of the printed specimen is 25 MPa. In tensile testing, the area below the stress-strain curve means the absorbed energy of the specimen before breakage. The absorbed energy is proportional to the toughness of the material. The absorbed energy of the filament during tensile testing is larger than the one of the printed specimens. Additionally, the maximum strain of the specimen in tensile testing before breakage would be an index of the ductile property of the material. The maximum strain of the extruded filament is about 32.2% in Figure 7, and one of the printed specimens is larger than 40% in Figure 7. The filament thus shows a lower Young’s modulus and ductile behavior. However, the A printed specimen with higher crystallinity would also produce a higher Young’s modulus. As shown in Table 1, the crystallinity of the printed specimen was greater than that of the filament, and crystallinity is one of the most important factors affecting yield strength. Serial testing of tensile strength was then carried out to derive an average yield strength of 24.65 MPa, as shown in Figure 8. The Young’s modulus of the filament shown in Figure 7 is 440.0 MPa, while that of the printed specimen is 481.57 MPa.

The crystallinity is, therefore, the reason for the changes observed in the Young’s modulus, although the lower molecular weight of PET extruded filament could also lead to a lower Young’s modulus. Moreover, talcum powder could improve the viscosity of melting PET during the filament extrusion process. Talcum powder would also enhance crystallinity, thereby yielding a significantly higher Young’s modulus. Hence, the Young’s modulus increases with the increase in crystallinity and the addition of talcum powder. The yield strength also increases with the increase in crystallinity and molecular weight of the material. Once the yield strength is enhanced, the ductile increases with the increase in molecular weight, and the maximum strain on breakage in tensile testing is negatively proportional to the PET crystallinity. Additionally, the maximum strain on breakage is positively proportional to the molecular weight of PET. The extruded filament presented very high elongation-at-break values with strain-at-break values of about 32%. The printed PET specimen had strain-at-break values of about 30%. The strain-at-break value is negatively proportional to the crystallinity of PET.

## 4. Conclusions

In this work, injection-molding-grade PET pellets for the extrusion of 3D printing filament were successfully derived under accurate control of the extrusion temperature. Extrusion can lead to chain scissions of PET, but the mechanical properties of the filament and printed specimen in this study were not significantly reduced due to the higher crystallinity provided by the addition of talcum powder. The non-isothermal crystallization analyses further highlighted the various mechanical properties. The main factors affecting the mechanical properties of the PET filament and printed specimen for virgin PET pellets were the decrease in molecular weight and the presence of talcum powder, which provided a higher Young’s modulus and improved yield stress. Conversely, ductility (with respect to the filament) decreased in the printed specimen for the same reason.

## Figures and Tables

**Table 1:** Calorimetric parameters for PET pellets, extruded filament, and printed specimen: crystallization temperature (*T*_C_), crystallization enthalpy (Δ*H*_C_), glass transition temperature (*T*_g_), melting temperature (*T*_m_), melting enthalpy (Δ*H*_m_), and degree of crystallinity (*X*_C_).

Material	Cooling Scan		Heating Scan			
	*T*_C_ (°C)	Δ*H*_C_ (J/g)	*T*_g_(°C)	*T*_m_(°C)	Δ*H*_m_ (J/g)	*X*_c_(%)
Pellets	171.9	27.98	82.24	246.6	27.57	19.69
Filament	185.4	31.33	80.46	249.3	30.08	21.49
Printed specimen	190.1	35.17	80.17	249.4	32.36	23.11

**Table 2:** The intrinsic viscosity (*η*) and average molecular weight ($M_{V}^{\;}$) of PET pellets, filaments, and 3D printed specimens.

Sample	*η* (dL g^−1^)	*M_V_* (g mol^−1^)
Pellets	0.710	2.012 × 10^4^
Filaments	0.695	1.961 × 10^4^
3D printed specimens	0.633	1.749 × 10^4^

**Table 3:** Decomposition temperatures and residual weights of the PET pellets, extruded filament, and 3D-printed materials.

Material	Onset Temperature (T_o_) °C	Peak Degradation Temperature (T_p_) °C	Residual Weight %
Pellets	384.88	425.55	0.015
Filament	386.93	402.42/428.24	6.601
Printed specimen	384.38	399.73/423.94	6.619

## Data Availability

All data generated or analyzed in this study are included in the published article.
